# Supplementary material for: Comparative genomics provides new insights into the diversity, physiology, and sexuality of the only industrially exploited tremellomycete: Phaffia rhodozyma
Source: BMC Genomics. 2016 Nov 9;17:901. doi: 10.1186/s12864-016-3244-7 (PMC5103461; doi:10.1186/s12864-016-3244-7)
Supplement: Additional file 6: — List of orphan genes with links to PFAM (related to Additional file 1: Table S1). (ZIP 1428 kb) [file 12864_2016_3244_MOESM6_ESM.zip › BLAST_HTML_FTR/G00561_P.html]

BLAST Search Results


```
BLASTP 2.2.27+


Reference:
Stephen F. Altschul, Thomas L. Madden, Alejandro A. Schäffer,
Jinghui Zhang, Zheng Zhang, Webb Miller, and David J. Lipman (1997),
"Gapped BLAST and PSI-BLAST: a new generation of protein database
search programs", Nucleic Acids Res. 25:3389-3402.


Reference for
composition-based statistics:
Alejandro A. Schäffer, L. Aravind, Thomas L. Madden, Sergei
Shavirin, John L. Spouge, Yuri I. Wolf, Eugene V. Koonin, and
Stephen F. Altschul (2001), "Improving the accuracy of PSI-BLAST
protein database searches with composition-based statistics and
other refinements", Nucleic Acids Res. 29:2994-3005.


Database: nr
           71,551,133 sequences; 26,053,659,533 total letters


Query= G00561_P

Length=352
                                                                      Score     E
Sequences producing significant alignments:                          (Bits)  Value

emb|CED83877.1|  hypothetical protein [Xanthophyllomyces dendrorh...   698    0.0  
ref|XP_007768547.1|  hypothetical protein CONPUDRAFT_153679 [Coni...  43.5    0.25 
ref|XP_007848179.1|  hypothetical protein Moror_4441 [Moniliophth...  41.6    1.3  
ref|WP_051555386.1|  hypothetical protein [Anaerovibrio lipolyticus]  40.4    2.4  
ref|WP_052211956.1|  hypothetical protein [Anaerovibrio lipolyticus]  40.4    2.5  
gb|EIE88010.1|  hypothetical protein RO3G_12721 [Rhizopus delemar...  40.0    4.1  


 >emb|CED83877.1| hypothetical protein [Xanthophyllomyces dendrorhous]
Length=351

 Score =  698 bits (1802),  Expect = 0.0, Method: Compositional matrix adjust.
 Identities = 351/351 (100%), Positives = 351/351 (100%), Gaps = 0/351 (0%)

Query  1    MCLLASSTELPPLIPAFNSLLLTLQIARPLDAPLTLRSFSPHLLLQTLSSTLRQALPDLP  60
            MCLLASSTELPPLIPAFNSLLLTLQIARPLDAPLTLRSFSPHLLLQTLSSTLRQALPDLP
Sbjct  1    MCLLASSTELPPLIPAFNSLLLTLQIARPLDAPLTLRSFSPHLLLQTLSSTLRQALPDLP  60

Query  61   PLPPLTDRSNAYIEKRREEIEVVKYILGILSFRVSAGFSDISPASVVDRDDDHLVVLARG  120
            PLPPLTDRSNAYIEKRREEIEVVKYILGILSFRVSAGFSDISPASVVDRDDDHLVVLARG
Sbjct  61   PLPPLTDRSNAYIEKRREEIEVVKYILGILSFRVSAGFSDISPASVVDRDDDHLVVLARG  120

Query  121  LILLALEEGWKLGEGSETESEGDREALPPGWDSAELETDKDRHNVLGRVRKEELYWKNKR  180
            LILLALEEGWKLGEGSETESEGDREALPPGWDSAELETDKDRHNVLGRVRKEELYWKNKR
Sbjct  121  LILLALEEGWKLGEGSETESEGDREALPPGWDSAELETDKDRHNVLGRVRKEELYWKNKR  180

Query  181  SRSPGRKSKNRRERWNKREAKTSLSLSESSGHDEDNEADGKDHSASHFSPAYSFRPLESP  240
            SRSPGRKSKNRRERWNKREAKTSLSLSESSGHDEDNEADGKDHSASHFSPAYSFRPLESP
Sbjct  181  SRSPGRKSKNRRERWNKREAKTSLSLSESSGHDEDNEADGKDHSASHFSPAYSFRPLESP  240

Query  241  PPSKLPRPITPTGSSPSKSVSRPSRRLSLSSCLSESCDFPLDRDLLTFSPAPEDVYALGP  300
            PPSKLPRPITPTGSSPSKSVSRPSRRLSLSSCLSESCDFPLDRDLLTFSPAPEDVYALGP
Sbjct  241  PPSKLPRPITPTGSSPSKSVSRPSRRLSLSSCLSESCDFPLDRDLLTFSPAPEDVYALGP  300

Query  301  SCPQDHVLIGLDPITKHTAPAEWLSNRKEEEIKLKDALDRLTDQTSGQTHT  351
            SCPQDHVLIGLDPITKHTAPAEWLSNRKEEEIKLKDALDRLTDQTSGQTHT
Sbjct  301  SCPQDHVLIGLDPITKHTAPAEWLSNRKEEEIKLKDALDRLTDQTSGQTHT  351


>ref|XP_007768547.1| hypothetical protein CONPUDRAFT_153679 [Coniophora puteana RWD-64-598 
SS2]
 gb|EIW81131.1| hypothetical protein CONPUDRAFT_153679 [Coniophora puteana RWD-64-598 
SS2]
Length=510

 Score = 43.5 bits (101),  Expect = 0.25, Method: Compositional matrix adjust.
 Identities = 38/126 (30%), Positives = 57/126 (45%), Gaps = 20/126 (16%)

Query  13   LIPAFNSLLLTLQIARPLDAPLTLRSFSPHLLLQTLSSTLRQALPDLPPLPPLTDRSNAY  72
            LI A N+LL+ L +   LD+P  L   +P LLL  L S +   L    PLPP    S + 
Sbjct  22   LIDALNNLLIALSLPFVLDSPTDL---TPSLLLAILESIIHARL----PLPPAIRASRSP  74

Query  73   IEKRREEIEVVKYILGILSFRV---------SAGFSDISPASVVDRDDDHLVVLARGLIL  123
            + +    +E +   LG+L   +         S G  ++ P  + + D D +V + R L  
Sbjct  75   VAR----VEAMHVFLGVLQCDLLDAKTDADASVGLDEVDPERLAEGDWDEVVFVGRLLCW  130

Query  124  LALEEG  129
            LA   G
Sbjct  131  LARRRG  136


>ref|XP_007848179.1| hypothetical protein Moror_4441 [Moniliophthora roreri MCA 2997]
 gb|ESK92519.1| hypothetical protein Moror_4441 [Moniliophthora roreri MCA 2997]
Length=655

 Score = 41.6 bits (96),  Expect = 1.3, Method: Compositional matrix adjust.
 Identities = 49/188 (26%), Positives = 79/188 (42%), Gaps = 25/188 (13%)

Query  130  WKLGEGSETESEGDREALPPGWDSAELETDKDRHNVLGRVRKEELYWKNKRSRSPGRKSK  189
            W +G GS + ++GD E L P     +++  K +  +L  V  E        +R  G  + 
Sbjct  460  WVVG-GSISVNDGDAEELSPENGLHKVKVKKGQTTLLVSVPAE----VTIENRPHGSVAV  514

Query  190  NR---RERWNKREAKTSLSLSESSGHDEDNEADGKDHSASHFSPAYSFRPLESPPPSKLP  246
            +R      ++    +  L +     H  D + D K+       P       ESPP  KLP
Sbjct  515  HRGPLNYAFDISRVEKVLKVDPRESHAIDLQYDAKEDWEYAIDPTTVTYHYESPPSGKLP  574

Query  247  RPITPTGSSPSKSVSRPSRRLSLSSCLSESCDFPLDRDLLTFSPAPEDVYALGPSCPQDH  306
             PI  +G SPS         +++++C  E   +PL  D    +P PE+     P+C   +
Sbjct  575  SPIFDSGLSPSS--------VTVTACRIE---WPLGGDTFAANP-PEN-----PTCIGPN  617

Query  307  VLIGLDPI  314
            V+I L P 
Sbjct  618  VIITLSPF  625


>ref|WP_051555386.1| hypothetical protein [Anaerovibrio lipolyticus]
Length=421

 Score = 40.4 bits (93),  Expect = 2.4, Method: Compositional matrix adjust.
 Identities = 37/129 (29%), Positives = 58/129 (45%), Gaps = 25/129 (19%)

Query  94   VSAGFSDISPASVVDRDDDHLVVLARGLILLALEEGWKLGEGSETESE-GDREALPPGWD  152
            VS  F DISP   +D+     +VLA GL +   E    +G G    S+ G    +P  + 
Sbjct  62   VSVNFQDISPREAIDK-----IVLASGLSISEREGTLIIGRGKNIVSQLGQVHVIPVAY-  115

Query  153  SAELETDKDRHNVLGRVRKEELYWKNKRSRSPGRKSKNRRERWNKREAKTSLSLSESSGH  212
              +LE  K         R+ ELY++NK    P + +KN+  +  K         SE++  
Sbjct  116  -GDLEELK---------REAELYFQNKYLTEPKKAAKNKETKSGKA--------SENAIS  157

Query  213  DEDNEADGK  221
            ++ N+  GK
Sbjct  158  NDINKESGK  166


>ref|WP_052211956.1| hypothetical protein [Anaerovibrio lipolyticus]
Length=421

 Score = 40.4 bits (93),  Expect = 2.5, Method: Compositional matrix adjust.
 Identities = 37/129 (29%), Positives = 58/129 (45%), Gaps = 25/129 (19%)

Query  94   VSAGFSDISPASVVDRDDDHLVVLARGLILLALEEGWKLGEGSETESE-GDREALPPGWD  152
            VS  F DISP   +D+     +VLA GL +   E    +G G    S+ G    +P  + 
Sbjct  62   VSVNFQDISPREAIDK-----IVLASGLSISEREGTLIIGRGKNIVSQLGQVHVIPVAY-  115

Query  153  SAELETDKDRHNVLGRVRKEELYWKNKRSRSPGRKSKNRRERWNKREAKTSLSLSESSGH  212
              +LE  K         R+ ELY++NK    P + +KN+  +  K         SE++  
Sbjct  116  -GDLEELK---------REAELYFQNKYLTEPKKAAKNKETKSGKA--------SENAIS  157

Query  213  DEDNEADGK  221
            ++ N+  GK
Sbjct  158  NDINKESGK  166


>gb|EIE88010.1| hypothetical protein RO3G_12721 [Rhizopus delemar RA 99-880]
Length=2000

 Score = 40.0 bits (92),  Expect = 4.1, Method: Compositional matrix adjust.
 Identities = 40/159 (25%), Positives = 64/159 (40%), Gaps = 24/159 (15%)

Query  172  EELYWKNKRSRSPGRKSKNRRERWNKREAKT--SLSLSESSGHDEDNEAD------GKDH  223
            EE YW+N+R +  GR  K     ++ R      +  +     H  D+E        G+  
Sbjct  106  EEEYWENQRGKQCGRLFKRGESVYHCRNCGFDDTCVMCSKCFHSTDHEGHDVKIWIGRGA  165

Query  224  SASHFSPAYSFR-----PLESPPPSKLPRPITPTGSSPSKSVSRPSRRLSLSSCLSESCD  278
                     +++      + S  PS    P  PT   P  SV  PS   S+ + + E+ +
Sbjct  166  GCCDCGDPEAWKVPLHCKIHSLDPSSTQIPNKPTQLKPLSSV--PS---SVLNSIQETIN  220

Query  279  FPLDRDLLTFSPAPEDVYALG------PSCPQDHVLIGL  311
              +D  L TF+ +PEDV ++G        C   H  +GL
Sbjct  221  VVMDYILETFAASPEDVMSIGNVDSIKKDCADSHQALGL  259


Lambda      K        H        a         alpha
   0.314    0.133    0.392    0.792     4.96 

Gapped
Lambda      K        H        a         alpha    sigma
   0.267   0.0410    0.140     1.90     42.6     43.6 

Effective search space used: 3065137128450


  Database: nr
    Posted date:  Sep 23, 2015 12:05 AM
  Number of letters in database: 26,053,659,533
  Number of sequences in database:  71,551,133


Matrix: BLOSUM62
Gap Penalties: Existence: 11, Extension: 1
Neighboring words threshold: 11
Window for multiple hits: 40
```
